# SHIELD: an integrative gene expression database for inner ear research

**DOI:** 10.1093/database/bav071

**Published:** 2015-07-23

**Authors:** Jun Shen, Déborah I. Scheffer, Kelvin Y. Kwan, David P. Corey

**Affiliations:** ^1^Department of Pathology, Brigham and Women’s Hospital,; ^2^Harvard Medical School Center for Hereditary Deafness,; ^3^Department of Neurobiology,; ^4^Department of Cell Biology and Neuroscience, Rutgers University, Piscataway, NJ 08854, USA and; ^5^Howard Hughes Medical Institute, Harvard Medical School, Boston, MA 02115, USA

## Abstract

The inner ear is a highly specialized mechanosensitive organ responsible for hearing and balance. Its small size and difficulty in harvesting sufficient tissue has hindered the progress of molecular studies. The protein components of mechanotransduction, the molecular biology of inner ear development and the genetic causes of many hereditary hearing and balance disorders remain largely unknown. Inner-ear gene expression data will help illuminate each of these areas. For over a decade, our laboratories and others have generated extensive sets of gene expression data for different cell types in the inner ear using various sample preparation methods and high-throughput genome-wide approaches. To facilitate the study of genes in the inner ear by efficient presentation of the accumulated data and to foster collaboration among investigators, we have developed the Shared Harvard Inner Ear Laboratory Database (SHIELD), an integrated resource that seeks to compile, organize and analyse the genomic, transcriptomic and proteomic knowledge of the inner ear. Five datasets are currently available. These datasets are combined in a relational database that integrates experimental data and annotations relevant to the inner ear. The SHIELD has a searchable web interface with two data retrieval options: viewing the gene pages online or downloading individual datasets as data tables. Each retrieved gene page shows the gene expression data and detailed gene information with hyperlinks to other online databases with up-to-date annotations. Downloadable data tables, for more convenient offline data analysis, are derived from publications and are current as of the time of publication. The SHIELD has made published and some unpublished data freely available to the public with the hope and expectation of accelerating discovery in the molecular biology of balance, hearing and deafness.

**Database URL:** https://shield.hms.harvard.edu

## Introduction

The inner ear is a delicate organ essential for hearing and balance. It contains both auditory and vestibular components. The cochlea senses auditory stimuli, and the saccule, utricle and three semicircular canals—each with an osseous ampulla—receive vestibular stimuli. The inner ear is encased in a bony structure that creates a labyrinth surrounding the soft tissue and makes tissue isolation difficult. In addition, many distinct types of cells are intermixed within the inner ear. They are mainly divided into neuronal ganglion cells, sensory hair cells and various kinds of supporting cells, and each set has multiple subtypes.

The inner ear develops from a simple otocyst during early embryogenesis. Many signaling pathways provide instructive cues that promote development and drive morphogenesis of the otocyst into the architecturally complex inner ear. Normal inner ear function depends on coordinated roles of distinct cell types. Many disorders and environmental insults affect the inner ear and cause hearing loss. Metabolic defects, mitochondrial disorders, congenital dysmorphology, other hereditary non-syndromic hearing loss, viral infection, aminoglycoside antibiotics and noise exposure are common causes of hearing loss in patients of all ages. Understanding the molecular mechanisms of inner ear development and of mechanotransduction will lead us to better approaches to the prevention and treatment of inner ear disorders.

High-throughput genotyping and sequencing technologies have enabled rapid discoveries of risk loci and DNA variants associated with human genetic disorders, including hearing loss and balance impairment (1). However, it remains challenging to pinpoint the causal genetic defects due to the lack of functional evidence. Genes specifically expressed in certain types of cells that serve specialized biological functions in the body likely contribute to the uniqueness of these cells. For example, hair cells in the inner ear are specialized receptors that transduce mechanical stimulation of their apical hair bundles, called stereocilia, to neurotransmitter release, which allows us to hear. Loss of hair cell function causes hearing loss. Therefore, knowing the cell-type–specific gene expression will facilitate an understanding of proteins mediating specialized function, will inform interpretation of genetic variants and will expedite the identification of novel disease genes and their roles in inner development and function.

Tremendous international effort such as the genotype-tissue expression project (GTEx) has been devoted to characterizing tissue-specific gene expression in many human tissue and cell types (2, 3). Unfortunately, the inner ear tissue is not included due to its inaccessibility and scarcity. Nevertheless, for over a decade, our laboratories and others have generated extensive sets of gene expression data for different cell types in the inner ear using various sample preparation methods and high-throughput genome-wide approaches (4–10). However, the data are scattered throughout the literature. It requires a significant amount of effort for researchers and clinicians to search, analyse and interpret the results to make full use of the valuable data. Here, we describe an integrative database of gene expression and annotation in the inner ear: the publicly accessible and extensively annotated Shared Harvard Inner Ear Laboratory Database (SHIELD; https://shield.hms.harvard.edu/). It serves as a portal to disseminate such data. We believe it will become a useful resource for interpreting variants in novel genes identified through genomic medicine for hearing and balance disorders.

## Database implementation

### System infrastructure

The SHIELD is an instance of a MySQL database running server version 5.1.49-3 on a Linux Debian system. The MySQL server is adjunct to the Orchestra high-performance computing cluster of Harvard Medical School (HMS) managed by the Research Computing Group of the HMS Information Technology Department. The Linux system also provides a web hosting service in Active/Active load balancing mode that allows for failover of web traffic in the event of a hardware or software error on one of the servers, with little to no user impact. The web infrastructure uses the Apache httpd web server.

**Figure 1.**
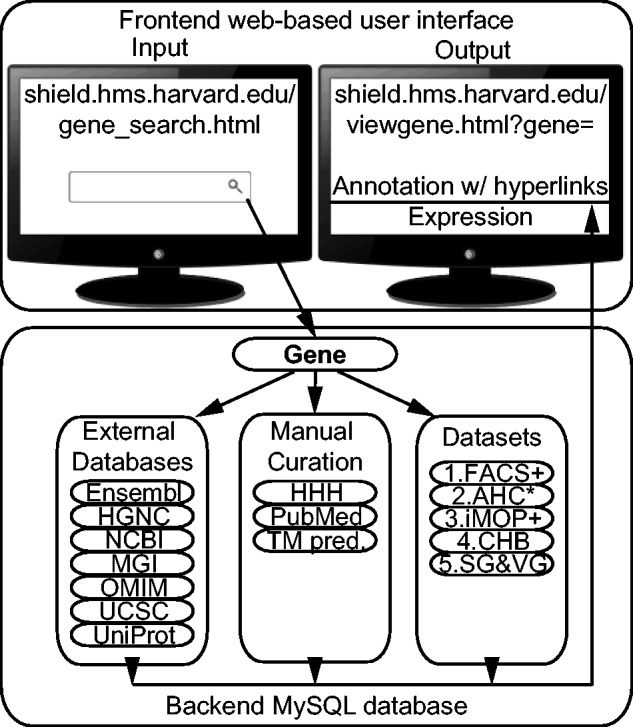
Architecture of the SHIELD. The URLs to external resources are HGNC, HUGO Gene Nomenclature Committee (http://www.genenames.org/); HHH, the Hereditary Hearing loss Homepage (http://hereditaryhearingloss.org/); NCBI, National Center for Biotechnology Information (http://www.ncbi.nlm.nih.gov/); MGI, Mouse Genome Informatics (http://www.informatics.jax.org/); OMIM Online Mendelian Inheritance in Man (http://www.omim.org/), UCSC, UCSC Genome Bioinformatics (https://genome.ucsc.edu/) and UniProt, the Universal Protein Resource (http://www.uniprot.org/). ‘TM pred.’ refers to manually annotated transmembrane domain prediction. Abbreviations for the datasets are explained in Table 1. ‘+’ and ‘*’ indicate unpublished data and unpublished statistical analysis available in the SHIELD. The line under ‘Annotation w/ hyperlinks’ represents clickable hyperlinks to other resources.

We designed and implemented the SHIELD as an integral system of a backend relational MySQL database instance and a front-end web user interface (Figure 1). The SHIELD consists of three types of contents: (i) annotation and curation of gene information; (ii) datasets of gene expression information of the inner ear and(iii) database access statistics. All information is converted to structured data stored in tables. Each table has a unique primary key. Related fields in other tables are linked as foreign keys. All primary keys and foreign keys are indexed for fast data retrieval. The web portal provides a publicly accessible user interface. The web pages are implemented using Personal Home Page Tools (PHP) and javascript scripting languages as well as cascading style sheets. The annotation and gene expression information are dynamically retrieved from the SHIELD using structured query language (SQL). The database and web contents are backed up daily.

### Annotations

Many public databases of gene information are available (11–16). However, different public databases often use different sets of unique identifiers (IDs) to describe the same genes or homologous genes in different species. One challenge of comparing large-scale biological datasets is the unification of gene names; otherwise, researchers spend a lot of effort in converting gene IDs when searching different databases. Another is the likelihood of missing some databases due to unfamiliarity; this is particularly true for clinicians and researchers who are specialized in inner ear research but are not necessarily familiar with genomics and bioinformatics. One goal of the SHIELD is to integrate relevant gene annotation information from various public databases in one centralized location.

For the SHIELD, annotations were derived from public databases and literature. Currently implemented annotations include official gene symbols, description of the gene name and synonyms, human and mouse chromosome cytogenetic banding, RefSeq RNA and protein (for protein coding genes) accession numbers, National Center for Biotechnology Information (NCBI) Entrez gene ID, genomic coordinates in both mouse reference genome assemblies mm9 and mm10, Ensembl, the Vertebrate Genome Annotation Database (VEGA) Mouse Genome Informatics, UniProt, Online Mendalian Inheritance in Man and gene ontology.

For each protein coding genes, we display all UniProt protein isoforms for that gene, the length in amino acid residues and the predicted number of transmembrane domains (TMs). We predicted TMs by running TMHMM2.0 run on all UniProt protein isoforms of each gene (17). The number of TMs is of special interest for research in sensory function, because many key proteins involved in signaling—such as the mechanotransduction ion channels—are integral membrane proteins. This information would help identify candidate genes for the components of the mechanotransduction apparatus of the inner ear.

We also performed manual curation of inner ear disorders including syndromic and non-syndromic hearing loss according to the Hereditary Hearing Loss Homepage (http://hereditaryhearingloss.org) and primary literature. The annotation shows whether a mouse gene or human homolog is associated with known inner ear disorders or falls within previously mapped genetic loci.

### Datasets

Gene expression datasets from five different studies are currently incorporated in the SHIELD. They represent a variety of model organism species, developmental stages, cell types, sample preparation techniques and data acquisition platforms (Table 1). A description of currently available datasets can be found at the ‘DATASETS’ tab on the SHIELD website. All five studies have been published. However, we have also included unpublished data and/or analyses for the first three datasets in the SHIELD.

**Table 1.** Comparison of the five datasets currently available in the SHIELD

Datasets	Organism	Developmental stages	Cell type	Organ component	Platform
1. FACS	Mouse	E16, P0, P4, P7, P16	hair cells and non-hair cells from the sensory epithelia	cochlea and utricle	RNAseq (3’DGE)
2. AHC	Mouse	P25-P40	inner hair cells and outer hair cells	cochlea	Microarray (GeneChip Mouse Gene 2.0 ST)
3. iMOP	Mouse	E12-E14	otosphere progenitors and iMOP	otocyst	RNAseq (full-length mRNA)
4. CHB	Chicken	E20-E21	stereocilia of hair cells	utricle	Mass spectrometry
5. SG&VG	Mouse	E12, E13, E16, P0, P6, P15	neurons	spiral ganglia and vestibular ganglia	Microarray (MOE430)

Abbreviations: AHC, adult hair cells; CHB, chicken hair bundle; DGE, digital gene expression, E embryonic; FACS, Fluorescence-activated cell sorting; iMOP, immortalized multipotent otic progenitor, P postnatal; SG, spiral ganglia and VG, vestibular ganglia.

The first and the most recently published dataset is called ‘Fluorescence-activated cell sorting (FACS)-Sorted Hair Cells—RNAseq’ (9). It is based on fluorescence activated cell sorting (FACS) of cells with different functions from inner-ear organs at discrete developmental stages. Cells were separately isolated from the inner ear sensory epithelia of genetically engineered mice that express green fluorescent protein driven by a hair-cell–specific promoter. Digital gene expression data were generated by deep sequencing of the 3’ end of cDNAs on the Illumina next generation sequencing platform. This dataset from the Corey laboratory provides a comprehensive catalog of genes expressed in the sensory hair cells and non-hair cells, in a hearing organ (the cochlea) and a balance organ (the utricle), from embryonic day 16 (E16) to postnatal days 0 (P0), P4 and P7. It thus allows the identification of genes that are preferentially expressed in specific cell types at discrete developmental stages. In addition to the published dataset (NCBI Gene Expression Omnibus (GEO) accession GSE60019), we also added unpublished gene expression results of hair cells and non-hair cells from P16 mouse utricles as well as a validation study with a biological duplicate of E16 mouse cochleae and utricles.

The second dataset, ‘Adult Cochlear Inner and Outer Hair Cells—GeneChip’, is based on P25 to P30 adult mice studied in the He laboratory (6). Individual inner and outer hair cells were dissociated and manually picked. This dataset further detailed the differences in gene expression of mature inner and outer hair cells. Total RNA expression profiles were generated using high-density mouse gene expression microarrays. We added additional statistical analysis of differential gene expression in the SHIELD on top of the published dataset (NCBI GEO accession GSE56866).

The third dataset is ‘Otic Progenitor Cells—RNAseq and ChIPseq’. This dataset, developed by Dr Kelvin Kwan, is derived from primary otosphere culture and immortalized multipotent otic progenitor cells (iMOP) treated with various growth factors (10). The iMOP cells are a continuously proliferating cell line of progenitors obtained from mouse embryonic cochleae. Different growth factor treatment schemes either maintain the proliferative potential of the iMOP cells or allow them to differentiate (10). The changes in gene expression under those different conditions are profiled by RNAseq of purified full-length mRNAs. In addition to the published dataset (NCBI GEO accession GSE62541), we also added unpublished data from iMOP cells treated with epidermal growth factor.

The fourth dataset, ‘Stereocilia Proteomics—Mass Spectrometry’, is derived from a proteomic study of chicken utricular stereocilia carried out in the Barr-Gillespie laboratory (5). Stereocilia proteins purified from E20 to E21 chicken utricles were identified by mass spectroscopy; the protein data are an important complement to transcriptomes, and are derived from just the sensory organelles of hair cells.

Finally, we incorporated an extensive microarray dataset from mouse spiral and vestibular ganglia, created by the Goodrich laboratory (4). E12 to P15 neurons in these ganglia relay hearing and balance signals to the brain, and a significant part of noise-induced hearing loss results from these neurons disconnecting from overdriven hair cells.

### Statistics of database access

The SHIELD has been open to the public since its launch in March 2012. It has been accessed over 550 000 times at an average of 426 requests per day. Seventy-three percent of the requests were searches for genes. The SHIELD keeps a count of each gene page being accessed, although lacking the ability to track the origin of the searches. Each of the top 10 most frequently searched-for genes has been queried more than 300 times (Table 2). These include four known human deafness genes *USH1C, CIB2, TMC1* and *MYO7A* (18–22); two known transcription factors, *ATOH1* and *SOX2*, that are essential in determining cell fate and regenerative potential in the inner ear (23, 24); and one new candidate gene for human deafness (*XIRP2*) (25, 26). Also among the top 10 are three transcription factors preferentially expressed in the hair cells that could be key regulators of hair cell development: *NEUROD6*, essential for neuronal fate determination in retina (27–29); *BARHL1*, controlled by ATOH1 and required for hair-cell survival (30); and *NHLH1*, a basic helix-loop-helix protein like ATOH1, implicated in neurogenesis (31).

**Table 2.** The 10 most frequently searched-for genes in the SHIELD as of 1 May 2015

Rank	Gene	Description	Number of times the gene page viewed	Articles about gene function in PubMed
1	*ATOH1*	atonal homolog 1	857	103
2	*USH1C*	Usher syndrome 1C	587	39
3	*CIB2*	calcium and integrin binding family member 2	555	10
4	*XIRP2*	xin actin-binding repeat containing 2	434	17
5	*NEUROD6*	neurogenic differentiation 6	392	13
6	*BARHL1*	BarH-like 1 (Drosophila)	387	10
7	*TMC1*	transmembrane channel-like gene family 1	364	39
8	*NHLH1*	nescient helix loop helix 1	352	13
9	*SOX2*	sex determining region Y-box 2	345	498
10	*MYO7A*	myosin VIIA	301	90

The genes are ranked by the times the gene page has been viewed. The number of articles about gene function in PubMed was based on (Search $gene[gene] AND alive[prop] NOT newentry[gene]) where $gene represents the gene name searched.

## Database usage

**Figure 2.**
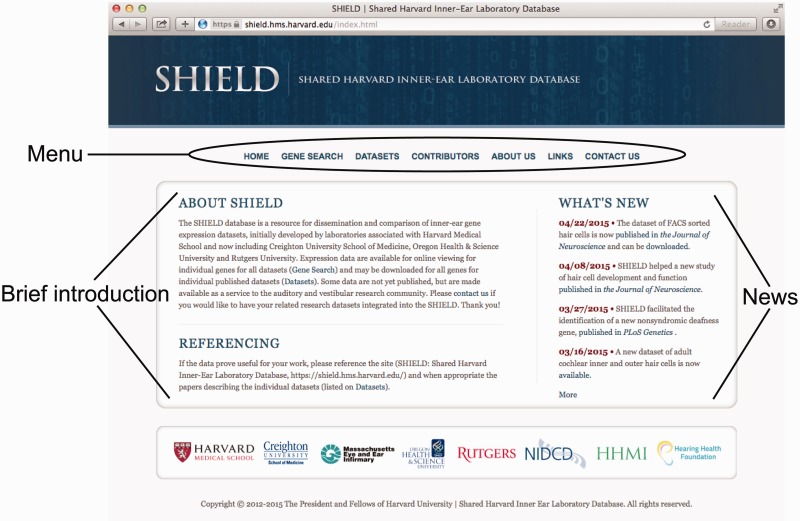
The Web Portal of the SHIELD. The homepage of the SHIELD shows the site logo, the menu, a brief introduction to the site, and news. At the bottom are the logos of the participating institutions and funding agencies.

The SHIELD is designed to provide integrated information about expression of inner ear genes, their roles in inner ear development and their association with hearing and balance disorders. Users can freely access the data in the SHIELD through a simple user-friendly interface (Figure 2). The home page presents a brief introduction to the database on the left side. The right side of the home page broadcasts news and announcements, constantly updated. The news and announcements are typically new publications that contributed data to the SHIELD or used data in it, as well as new features, enhancements and bug fixes of the database. Clicking ‘More’ at the bottom of the ‘WHAT’S NEW’ list will bring up archived news information. The top menu items include ‘HOME’, ‘GENE SEARCH’, ‘DATASETS’, ‘CONTRIBUTORS’, ‘ABOUT US’, ‘LINKS’ and ‘CONTACT US’. Users can click any of the menu items to use the database or get more information. No password or user registration is required to use the database, although the ‘CONTACT US’ tab offers a feedback form for any user who wishes to ask questions or leave comments. We expect that user feedback will continuously help improve the accuracy of the annotations and their relevance to the inner ear.

### Search

The most useful feature of the SHIELD is the search function. Users can search the database in two ways. First, users can choose ‘GENE SEARCH’ in the menu. This will bring up the GENE SEARCH page, including a simple web form of a text input field with a magnifying glass icon. Users can simply type the search term in the input field and hit the ‘enter/return’ key on their keyboard. The search term may be a gene name or a partial gene name. The input terms are parsed to validated strings, and the web engine executes dynamically generated SQL SELECT queries to search the database. If the search result returns a single exact match, the page will refresh to display the information of that gene. If there are two to 100 matches of genes, the number of matches and a list of matched genes names with hyperlinks to the gene-view pages will be displayed. If there are more than 100 matches, the total number of matches and only the first 100 matches will be displayed. In this case, we recommend users to refine the search term. Importantly, the search space not only includes currently approved official mouse and human gene names but also old names, alias and synonyms. Because gene names are constantly evolving, multiple annotation tables are searched for relevant information on the input term. The second method provides a shortcut to search through a direct URL. Users can simply append the search term to the end of ‘https://shield.hms.harvard.edu/viewgene.html?gene=’ and hit the ‘enter/return’ key on their keyboard. This has essentially the same effect as the search form, but it is more efficient because it bypasses the form.

### View

Users can view detailed annotations and inner ear expression information in a gene-centric manner by clicking a gene in the search results. The gene-view page is automatically loaded, if the search returns a unique gene. The top-level menu and the gene name are at fixed positions. The gene name is hyperlinked to the GeneCards page of the gene (32). The information panel is scrollable, which accommodates expanding knowledge. The annotations are displayed first in the information panel. The [PubMed] link on the first line brings users directly to PubMed search of the gene. Entrez ID is hyperlinked to the NCBI Gene, and genomic coordinates to the University of California Santa Cruz (UCSC) Genome Browser. Other IDs and descriptions such as Ensembl, VEGA, Mouse Genome Informatics, UniProt, mouse alleles and Online Mendelian Inheritance in Man diseases are all hyperlinked to respective databases. Following the annotations are sections for gene expression, one from each study as described in the DATASETS tab. Users can hover the mouse pointer over ‘Chart’ in the ‘FACS-Sorted Hair Cells’ section to view the graph of cell-type–specific gene expression of the gene in cochlea and utricle at four distinct ages. Normalized data, fold change and statistical significance are displayed for each gene.

### Download

In addition to using the search function, users also have the option to download individual datasets of published studies included in the SHIELD. Users can go to the DATASETS tab. Following the description of each study is the publication to cite. Users can click the link with the downward arrow adjacent to it to download the data files in either Excel or tab delimited plain text format. The raw data have been deposited in the NCBI Gene Expression Omnibus and can directly be accessed by clicking the GEO accession number.

### Examples of research use

The integration of different inner-ear datasets and links to a variety of other database makes the SHIELD a useful starting point for inner ear research. For example, we identified genes preferentially expressed in hair cells or non-hair cells in the sensory epithelia of the inner ear. Among the most differentially expressed genes, we found that homologs of established human hearing loss genes are highly enriched. Thirty-three of the 72 well-established hearing loss genes are differentially expressed by at least 2-fold with a false discovery rate (FDR) of <0.1, but only 6.8% of all genes meet the same criteria (odds ratio = 6.6, 95% confidence interval 4.3–10.0, *Z* = 8.9, *P* < 0.0001). This suggests that the likelihood of a gene’s impact on inner ear function can be estimated based on the degree (fold change) and statistical significance (FDR) of the differential expression in hair cells versus non-hair cells.

The SHIELD can support the identification of deafness genes discovered by other means. For example, the *SYNE4* gene has been proposed as a human deafness gene (33). However, only a single disease allele has been reported. The SHIELD shows that the gene is expressed 11-fold higher in hair cells with a FDR of 1.56 × 10^−^^4^, supporting an important role in normal hearing and the likelihood that pathogenic variants in *SYNE4* cause loss. Similarly, the *ESRRG* gene has been associated with hearing function in a genome-wide association study, but the association did not quite reach the genome-wide significance (34). This gene is expressed 4.6 times higher in hair cells (FDR < 0.01), again supporting a specific role in hair cell function. Indeed, recently published studies have illustrated the utility of the SHIELD in discovery of hearing-loss genes (19, 35).

The SHIELD can also help elucidate the molecular mechanism of hearing and balance by integrating information from different datasets. From the first dataset, for example, we see that the *Xirp2* gene is highly expressed in the mouse inner ear, almost exclusively in hair cells (26-folds enrichment, FDR 1.47 × 10^−14^), and preferentially in postnatal compared with embryonic hair cells (12-fold enrichment, FDR 5.72 × 10^−^^6^). The third dataset shows its low expression in progenitor cells, consistent with its expression rising postnatally. The fourth dataset indicates that the XIRP2 protein is concentrated in steoreocilia by about 13-fold compared with the cell body, and that each setreocillium has more than 4000 XIPR2 proteins. All these data suggest that XIRP2 plays an important role in the unique function of hair cell stereocilia. The SHIELD annotation shows that it is not a transmembrane protein, but it has multiple actin-binding domains suggesting interaction with the actin cores of stereocilia. The ‘mouse allele’ annotation shows that several mouse models have already been made, so it may be relatively straightforward to investigate its role. In addition, the ‘deafness gene and locus’ annotations indicate that human *XIRP2* falls within two different but overlapping mapped deafness loci DFNB27 and DFNA16. Such information prompted two research groups to investigate the role of XIRP2 in the inner ear, revealing its function in maintaining the paracrystalline actin filament array of stereocilia (25, 26).

## Conclusion and future directions

In conclusion, we have developed the SHIELD, an integrated resource that compiles, organizes and analyses much of the current genomic, transcriptomic and proteomic knowledge of the inner ear. Currently, the SHIELD provides the auditory and vestibular research community with downloadable data files for published datasets and a searchable user interface to view individual genes. The gene-centric view of each gene integrates annotation from various other databases, cross-references among different species and displays high-quality high-throughput transcriptomic and proteomic gene expression data of the inner ear with visualization through a freely accessible public online portal. Since its official launch in March 2012, the SHIELD website has contributed to new gene discovery and functional confirmation for a number of studies (10, 19, 25, 26, 35–49).

In the coming years, we will continue to optimize the structure, content and user interface of the SHIELD. We will synchronize the SHIELD with other public databases to maintain updated annotations. Furthermore, we will expand the database by adding new datasets of the inner ear gene expression for additional specific cell types, developmental stages, experimental conditions and different species as they become available. In addition, we will implement advanced search options to allow batch retrieval and online filtering of the data. We expect the database will become the one-stop resource for inner ear molecular and genetic research.
